# Longitudinal associations between β-amyloid and cortical thickness in mild cognitive impairment

**DOI:** 10.1093/braincomms/fcad192

**Published:** 2023-07-04

**Authors:** Elijah Mak, Liwen Zhang, Chin Hong Tan, Anthonin Reilhac, Hee Youn Shim, Marcus Ong Qin Wen, Zi Xuen Wong, Eddie Jun Yi Chong, Xin Xu, Mary Stephenson, Narayanaswamy Venketasubramanian, Juan Helen Zhou, John T O’Brien, Christopher Li-Hsian Chen

**Affiliations:** Department of Psychiatry, University of Cambridge, Cambridge, CB2 2QQ, United Kingdom; Memory Aging and Cognition Centre, Department of Pharmacology, Yong Loo Lin School of Medicine, National University of Singapore, Singapore, 119228, Singapore; Centre for Sleep and Cognition, Yong Loo Lin School of Medicine, National University of Singapore, Singapore, 119228, Singapore; Division of Psychology, Nanyang Technological University, Singapore, 637331, Singapore; Lee Kong Chian School of Medicine, Nanyang Technological University, Singapore, 308232, Singapore; Clinical Imaging Research Centre, the Agency for Science, Technology and Research, and National University of Singapore, Singapore, 117599, Singapore; Memory Aging and Cognition Centre, Department of Pharmacology, Yong Loo Lin School of Medicine, National University of Singapore, Singapore, 119228, Singapore; Centre for Sleep and Cognition, Yong Loo Lin School of Medicine, National University of Singapore, Singapore, 119228, Singapore; Memory Aging and Cognition Centre, Department of Pharmacology, Yong Loo Lin School of Medicine, National University of Singapore, Singapore, 119228, Singapore; Department of Psychological Medicine, Yong Loo Lin School of Medicine, National University of Singapore, Singapore, 119228, Singapore; Memory Aging and Cognition Centre, Department of Pharmacology, Yong Loo Lin School of Medicine, National University of Singapore, Singapore, 119228, Singapore; Department of Psychological Medicine, Yong Loo Lin School of Medicine, National University of Singapore, Singapore, 119228, Singapore; Memory Aging and Cognition Centre, Department of Pharmacology, Yong Loo Lin School of Medicine, National University of Singapore, Singapore, 119228, Singapore; School of Public Health, and the 2nd Affiliated Hospital, Zhejiang University School of Medicine, Hangzhou, Zhejiang, 311100, China; Centre for Translational MR Research (TMR), Yong Loo Lin School of Medicine, National University of Singapore, Singapore, 117549, Singapore; Raffles Neuroscience Centre, Raffles Hospital, Singapore, 188770, Singapore; Centre for Sleep and Cognition, Yong Loo Lin School of Medicine, National University of Singapore, Singapore, 119228, Singapore; Centre for Translational MR Research (TMR), Yong Loo Lin School of Medicine, National University of Singapore, Singapore, 117549, Singapore; Department of Electrical and Computer Engineering, National University of Singapore, Singapore, 119077, Singapore; Department of Psychiatry, University of Cambridge, Cambridge, CB2 2QQ, United Kingdom; Memory Aging and Cognition Centre, Department of Pharmacology, Yong Loo Lin School of Medicine, National University of Singapore, Singapore, 119228, Singapore; Department of Psychological Medicine, Yong Loo Lin School of Medicine, National University of Singapore, Singapore, 119228, Singapore

**Keywords:** regional β-amyloid, [^11^C]-PiB PET, cortical thickness, longitudinal cortical thinning, mild cognitive impairment

## Abstract

How beta-amyloid accumulation influences brain atrophy in Alzheimer's disease remains contentious with conflicting findings. We aimed to elucidate the correlations of regional longitudinal atrophy with cross-sectional regional and global amyloid in individuals with mild cognitive impairment and no cognitive impairment. We hypothesized that greater cortical thinning over time correlated with greater amyloid deposition, particularly within Alzheimer’s disease characteristic regions in mild cognitive impairment, and weaker or no correlations in those with no cognitive impairment. 45 patients with mild cognitive impairment and 12 controls underwent a cross-sectional [^11^C]-Pittsburgh Compound B PET and two retrospective longitudinal structural imaging (follow-up: 23.65 ± 2.04 months) to assess global/regional amyloid and regional cortical thickness, respectively. Separate linear mixed models were constructed to evaluate relationships of either global or regional amyloid with regional cortical thinning longitudinally. In patients with mild cognitive impairment, regional amyloid in the right banks of the superior temporal sulcus was associated with longitudinal cortical thinning in the right medial orbitofrontal cortex (*P* = 0.04 after False Discovery Rate correction). In the mild cognitive impairment group, greater right banks amyloid burden and less cortical thickness in the right medial orbitofrontal cortex showed greater visual and verbal memory decline over time, which was not observed in controls. Global amyloid was not associated with longitudinal cortical thinning in any locations in either group. Our findings indicate an increasing influence of amyloid on neurodegeneration and memory along the preclinical to prodromal spectrum. Future multimodal studies that include additional biomarkers will be well-suited to delineate the interplay between various pathological processes and amyloid and memory decline, as well as clarify their additive or independent effects along the disease deterioration.

## Teaser

Mak *et al*., demonstrated that baseline regional deposition in the temporal lobe was correlated with accelerated cortical thinning in individuals with mild cognitive impairment. Moreover, those with a combination of increased amyloid deposition and cortical thinning showed greater visual and verbal memory decline over time.

## Graphical Abstract

**Graphical Abstract:**
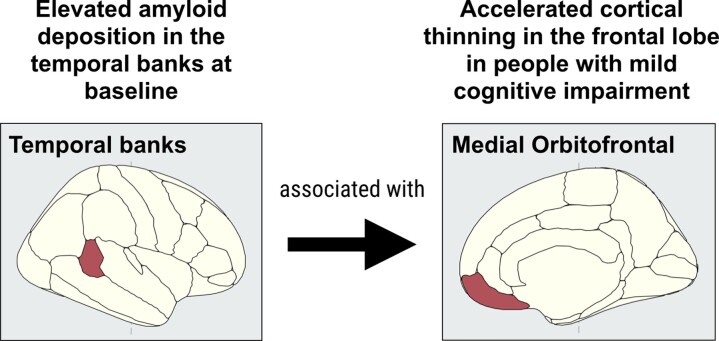

## Introduction

Brain atrophy is frequently reported in patients with Alzheimer's disease (AD). Decades prior to symptom manifestation beta-amyloid (Aβ) deposition in AD spreads from the neocortex to other brain areas as the disease progresses.^1^ Patients with AD dementia have shown relationships between higher levels of Aβ burden and greater atrophy in a number of cortical areas (e.g. precuneus, parietal, lateral frontal, and temporal),^2,3^ supporting the concept that Aβ accumulation predates brain structural abnormalities by several decades.^4,5^ Given the prolonged and ‘silent’ progr­ession of preclinical AD, there is a growing consensus that interventions may be more effective before the onset of symptoms and irreversible significant brain atrophy.^6^ Consequently, there is an urgent need for research on individuals with mild cognitive impairment (MCI), many of whom have increased Aβ and neurodegeneration at varying degrees^4,5,7^ and are at a higher risk of developing AD.^8^

Delineating some of the earliest brain alterations associated with incipient Aβ accumulation during the prodromal phase has the potential to aid in early detection and monitoring of disease progression. Previous studies of MCI that investigate the relationship between Aβ load and brain atrophy have yielded mixed results. Two widely used measurements of brain atrophy are brain volume and cortical thickness. One early study^9^ investigated the cross-sectional relationship between global Aβ burden and total grey matter volume, as well as the local association between regional Aβ and volume from the same area, and found no Aβ-related grey matter volume loss in MCI. In contrast, another study^10^ showed that greater regional Aβ burden at baseline was associated with a faster rate of grey matter volume decline over one year in MCI, primarily between Aβ in the precuneus/posterior cingulate cortex and volume decline in spatially distributed AD signature regions (e.g. medial temporal lobe and frontal regions). In addition, cortical thickness has been proposed as an alternative to grey matter volume^11^ for the assessment of AD-related brain atrophy, since evidence^12^ indicates that it is less impacted by potential confounders (e.g. total intracranial volume), has good ability in discriminating AD/MCI from controls, robust reliability on repeated scans, and strong correlation with AD pathology. There have been inconclusive findings about Aβ-associated cortical thinning. Briefly, cortical thinning in the precuneus has been associated with a higher cross-sectional global Aβ load in MCI.^13^ Another cross-sectional investigation that included both MCI and cognitively-normal elderly discovered global Aβ -associated cortical thinning in the precuneus, which mediated the relationship between Aβ and episodic memory.^14^ In contrast, Ye and colleagues^15^ found no cortical thinning in Aβ positive MCI versus Aβ negative MCI. While methodological differences may account some of these discrepant results, it is conceivable that Aβ has only mild to modest effects on brain atrophy in a regionally-selective manner, especially during the prodromal phase of AD.

Notably, Aβ deposition is distributed heterogeneously across cortical regions.^16^ Previous research has largely focused on global Aβ burden, which may diminish statistical ability to detect its early effects on brain atrophy. Furthermore, whereas most research used a cross-sectional design and showed conflicting results ,^9,13,15^ a longitudinal approach may be more sensitive and help clarify the Aβ-associated atrophy trajectory in MCI. To date, only one early longitudinal study has examined the relationship between regional Aβ burden and regional GMV across the whole brain in MCI.^10^ The findings, however, were derived from low resolution structural imaging (1.5 T) and regional brain volumes rather than cortical thickness.

We aimed to investigate the association of retrospective longitudinal cortical thinning on serial structural MRI with cross-sectional Aβ burden as measured by [^11^C]-PIB PET in MCI and NCI. We tested the association between global Aβ load and progressive regional cortical thinning. Because Aβ accumulation is heterogeneous across cortical regions, using global Aβ load would dilute potential Aβ effects on cortical thinning when Aβ predominantly deposits locally at the early disease stages. Thus, we *a priori* selected regions of interest (ROIs) from previous literature that have been identified as sites of early Aβ accumulation to determine whether regional Aβ load in each of those ROIs was longitudinally associated with regional cortical thinning. We hypothesised that MCI patients with a higher global and/or regional Aβ burden would have greater longitudinal cortical thinning, most notably in a spatial pattern resembling the cortical signature of AD neurodegeneration, whereas NCI would have less or no associations.

## Materials and methods

### Participants

We studied 45 clinically diagnosed MCI participants (age 71.8 ± 7.1 years, 48.9% female) and a demographically matched control group of 12 NCI, recruited from memory clinics and the community in Singapore as described previously.^17^ Diagnosis was made at consensus meetings between neurologists, research personnel, and psychologists based on clinical observations, brain imaging scans (MRI and/or CT), neuropsychological assessments and laboratory tests. Clinical diagnosis of MCI was made if participants 1) had subjective and objective cognitive impairment in at least one cognitive domain of the locally validated neuropsychological assessment battery (section *2.2. Neuropsychological testing*), and 2) were not demented and remained functionally independent. All NCI had a Mini-Mental State Examination (MMSE) score ≥ 26. Exclusion criteria included: 1) comorbidity with epilepsy or neuropsychiatric disorders (e.g. schizophrenia, bipolar disorder) associated with cognitive impairment; 2) disorders that may affect the central nervous system based on the Diagnostic and Statistical Manual of Mental Disorders (DSM-IV) (e.g. substance abuse disorder, toxic, nutritional or traumatic disorder); 3) central nervous system infections (e.g. viral encephalitis, syphilis, fungi, Creutzfield-Jacob disease, bacterium, tuberculosis or rickettsiae); 4) intracranial space occupying lesions (e.g. tumor); 5) intracerebral hemorrhage, leading to potential cognitive impairment; 6) hypoxic/anoxic, hypo-/hyper-tensive, uremic or hepatic encephalopathy; 7) moyamoya disease, cranial arteritis, or inflammatory vasculitides within the central nervous system; 8) obstructive or normal pressure hydrocephalus; and 9) MRI-incompatibilities, such as pregnancy or mental implants. Participant characteristics are reported in Table 1. This study was approved by the National Healthcare Group Domain-Specific Review Board. Written informed consent was obtained from all participants.

**Table 1:** Participant characteristics of participants

	MCI (*N* = 45)	NCI (*N* = 12)	Test statistic, *P*
Age (years)	74.5 (6.44)	76.1 (5.79)	t = 0.83, *P* = 0.419
Sex (% Female)	22 (48.9%)	9 (75%)	χ^2^ = 1.66, *P* = 0.198
Education (years)	8.07 (4.33)	8.00 (4.55)	t = 0.05, *P* = 0.964
Handedness, R/L	44/1	11/1	χ^2^ = 1.05, *P* = 0.38
APOE-ε4 carriers	14 (31.1%)	1(8.3%)	χ^2^ = 2.53, *P* = 0.162
MMSE	24.4 (3.91)	27.5 (2.21)	t = 3.46, *P* = 0.002*

Mean (SD) values are reported for continuous variables, unless otherwise indicated at the time of PET scanning.

Abbreviations: CDR, Clinical Dementia Rating, MCI, mild cognitive impairment, MMSE, Mini-Mental State Examination, NCI, no cognitive impairment.

* *P* < 0.05.

### Neuropsychological testing

All participants underwent neuropsychological testing, including the MMSE, Clinical Dementia Rating Scale and a cognitive assessment battery. The cognitive assessment battery consisted of seven cognitive domains, including verbal and visual memory, executive function, language, attention, visuomotor speed and visuoconstruction. Standardized domain z-scores were calculated as described previously^18,19^

### Image acquisition

Whole-brain T1-weighted structural MRI images were collected in a 3T Siemens Magnetom Tim Trio scanner (32-channel head coil) at baseline and year 2. The Magnetization prepared rapid gradient recalled echo (MPRAGE) sequence was applied (192 sagittal slices, slice thickness of 1 mm, isotropic voxel size of 1 mm^3^, TR = 2300 ms, TE = 1.9 ms, TI = 900 ms, FOV = 256 × 256 mm^2^, Flip angle of 9°). PET data were acquired from a Siemens Biograph mMR scanner^20^ at the Clinical Imaging Research Centre, National University of Singapore (Singapore). All participants received 370 MBq ^11^C-PIB injection, followed by 40∼70 min post-injection static PET imaging (176 axial slices, slice thickness of 1 mm, isotropic voxel size of 1 mm^3^, FOV = 256 × 256 mm^2^). The PET scan was performed at one time point (not necessarily at baseline) during the whole ongoing longitudinal follow-up (Supplementary Fig. 1). Given the gradual accumulation of Aβ deposition,^21^ we utilized the baseline and year 2 structural MRI images for atrophy evaluation irrespective of PET scan timing to increase the sample size.^17^ The average interval between the PET scan and the baseline MRI scan was 33.3 ± 15.9 months, and average interval between baseline and year 2 MRI scans was 23.6 ± 2.0 months.

### Image processing

#### Structural MRI pre-processing

Prior to pre-processing, structural MRI images first underwent visual inspection for motion artifacts. After that, pre-processing was performed, using the FreeSurfer pipeline (version 6.0).^22,23^ An additional FLAIR image was added to facilitate cortical surface reconstruction. Manual editing was performed where appropriate without diagnostic information. Cortical thickness was indexed as the shortest distance between the reconstructed white matter and pial surfaces. Accordingly, cortical thickness values were extracted from 68 cortical regions based on the Desikan-Killiany atlas.^11,24^ Participants were a subset of the cohort utilised in our previous study,^17^ and all passed image quality control.

#### PET pre-processing

Before tomographic image reconstruction, motion correction was first conducted, using the in-house developed rebinner with rigid motion correction.^25^ Estimation of motion parameter was performed every 20 s. After motion correction, images were reconstructed into a single static frame (voxel size = 2.09 × 2.09 × 2.03 mm^3^, voxel number = 344 × 344 × 344 voxels), applying a 3D Ordinary Poisson Ordered-subsets Expectation Maximization (OP-OSEM) algorithm (with all corrections performed including resolution modelling)^26^ and using three iterations and 21 subsets. Subsequently, the PetSurfer pipeline was applied for PET data pre-processing,^27^ including: 1) generating a high-resolution segmentation based on the pre-processed structural MRI image (see *Structural MRI pre-processing*); 2) co-registering the single static PET frame to the obtained high-resolution structural segmentation; 3) applying partial volume correction (PVC) with the symmetric geometric transfer matrix; and 4) calculating standardized uptake value ratio (SUVR) for each ROI as parcellated for the pre-processed structural MRI image (native space), with the cerebellar grey matter being the reference region. This resulted in 68 regional SUVR scores. Moreover, a global SUVR score was also obtained by averaging SUVR scores across four large brain regions, including the frontal, lateral temporal, lateral parietal and cingulate cortex.^28^

### Statistical analyses

#### Examining regional progressive cortical thinning in association with Aβ deposition

We used linear mixed models (R package ‘lme4’) to examine retrospective trajectories of longitudinal cortical thinning in association with cross-sectional Aβ burden, and used bootstrap resampling (*n* = 10 000) to estimate the 95% confidence interval (CI) of the model coefficients (R package ‘lmeresampler’). Specifically, we first determined whether global Aβ deposition affected cortical thinning in each of the 68 cortical ROIs over time (see Supplementary Fig. 2 for the visualization of cortical parcellations). Considering heterogeneous Aβ deposition across cortical regions, we then *a priori* selected ROIs from the literature that have been shown to be sites with the earliest Aβ deposition, and examined whether regional Aβ within each of these ROIs had local and/or local-to-distributed associations with any regional cortical thinning longitudinally (Model 1). These *a priori* selected ROIs (*n* = 8) included bilateral banks of the superior temporal sulcus, precuneus, posterior cingulate cortex and medial orbitofrontal cortex.^29,30^


(*Model* 1) $${\mathrm{Thickness}}_{\mathrm{ij}}=\beta 0+\beta 1^{*}A\beta_{i}+\beta 2^{*}{\mathrm{Time}}_{\mathrm{ij}}+\beta 3^{*}A\beta_{i}{\;}^{*}{\mathrm{Time}}_{\mathrm{ij}}+\beta 4^{*}{\mathrm{Age}}_{i}+b0_{i}+\varepsilon_{\mathrm{ij}}$$

In this model, Thickness_ij_ represented each regional cortical thickness for participant i at time j, time indexed the interval between baseline and the second MRI scan (baseline = 0), Aβ_i_ referred to cross-sectional either regional or global Aβ burden for participant i. β represented estimates for fixed effects (i.e. Aβ, time, interaction between Aβ and time, and age) and b denoted estimates for a random effect (i.e. individual intercept). Linear mixed models were built for MCI and NCI separately. Threshold was set at P_FDR_ < 0.05, applying false discovery rate (FDR) to correct for multiple testing (*n* = 68 cortical ROIs).

#### Relationships between Aβ, cortical thinning and cognitive decline

We used linear mixed models and bootstrap resampling to test clinical correlations between Aβ-associated cortical thinning and cognitive impairment. We first determined if and which cognitive domains showed decline over time (Model 2), applying FDR correction for the total number of cognitive domains evaluated (*n* = 7). Then we examined whether there was an interaction between the Aβ and regional cortical thinning in terms of predicting cognitive declines (Model 3).


(*Model* 2) $${\mathrm{cognition}}_{\mathrm{ij}}=\beta 0+\beta 1^{*}{\mathrm{Time}}_{\mathrm{ij}}+\beta 2^{*}{\mathrm{Age}}_{i}+b0_{i}+\varepsilon_{\mathrm{ij}}$$

(*Model* 3) $${\mathrm{cognition}}_{\mathrm{ij}}=\beta 0+\beta 1^{*}A\beta_{i}+\beta 2^{*}{\mathrm{Thickness}}_{\mathrm{ij}}+\beta 3^{*}A\beta_{i}{\;}^{*}{\mathrm{Thickness}}_{\mathrm{ij}}+\beta 4^{*}{\mathrm{Age}}_{i}+b0_{i}+\varepsilon_{\mathrm{ij}}$$

In Model 2, cognition_ij_ represented each of the seven cognitive domains for participant i at time j. In Model 3, cognition_ij_ referred to cognition within specific domains that showed longitudinal declines as identified in Model 2, Aβ and Thickness indexed specific Aβ burden and associated regional cortical thinning as identified in Model 1. Denotations for other parameters were the same as parameters in Model 1.

## Results

### Associations between Aβ burden and longitudinal cortical thinning

**Figure 1:**
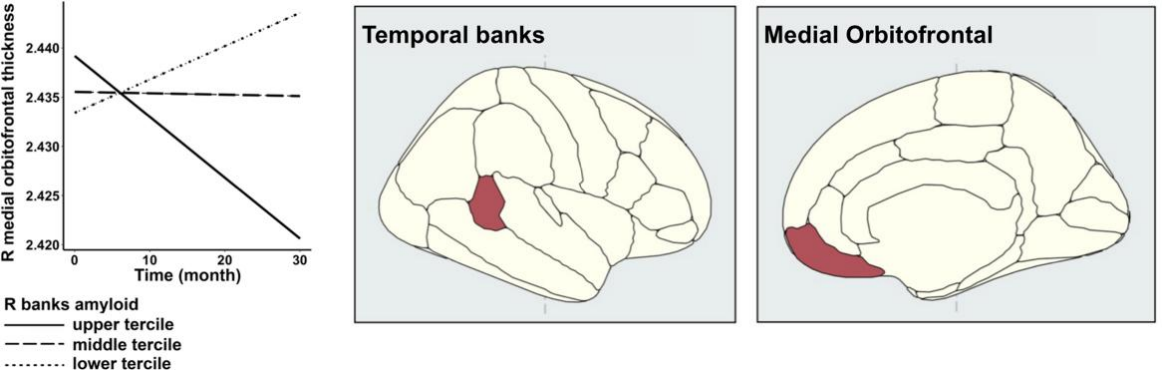
**Association between baseline Aβ deposition and longitudinal cortical thinning.** Greater Aβ burden in the banks of the right superior temporal sulcus is associated with faster rates of cortical thinning in the right medial orbitofrontal cortex in MCI (*N* = 45, linear mixed models: β = −0.0013, t(43) = −3.71, P_FDR_ = 0.04). Abbreviation: Aβ, amyloid deposition; MCI, mild cognitive impairment; R, right.

In MCI, more Aβ deposition in the banks of the right superior temporal sulcus was associated with faster longitudinal cortical thinning in the right medial orbitofrontal cortex (Fig. 1; β for Aβ * time interaction = −0.0013, t(43) = −3.71, P_FDR_ = 0.04). Bootstrapped linear mixed model also indicated significant Aβ * time interaction effect (95% CI = −0.0019 ∼ −0.0006). In contrast, NCI revealed no correlations between regional Aβ of interest and longitudinal cortical thinning in any of the regions studied. Using the global Aβ load, we found no significant relationships between Aβ and regional cortical thinning over time in NCI or MCI.

### Relationships between Aβ, cortical thinning and cognitive decline in MCI

**Figure 2:**
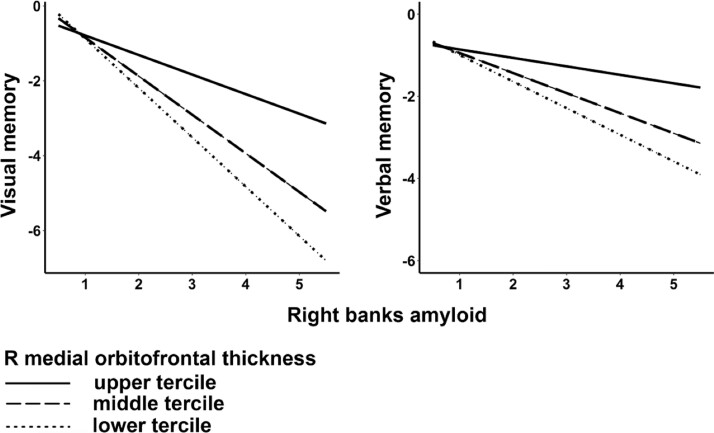
**Clinical relevance of elevated Aβ deposition and cortical thinning in MCI.** Higher Aβ burden in the right banks of the superior temporal sulcus and greater cortical thinning in the right medial orbitofrontal cortex were associated with faster visual memory decline in MCI (*N* = 45, linear mixed models: β = 3.18, t(50.8) = 2.79, *P* = 0.007) and verbal memory (*N* = 45, linear mixed models: β = 1.77, t(51.4) = 2.05, *P* = 0.045). Abbreviation: Aβ, amyloid deposition; MCI, mild cognitive impairment; R, right.

The MCI group showed longitudinal declines in both visual memory (β for time = −0.035, t(34.9) = −3.42, P_FDR_ = 0.006) and verbal memory (β for time = −0.027, t(37.8) = −3.43, P_FDR_ = 0.006). Bootstrap resampling showed further support for significant declines in visual memory (95% CI = −0.055 ∼ −0.015) and verbal memory (95% CI = −0.043 ∼ −0.012). We used linear mixed models to explore whether these memory declines in MCI were affected by Aβ in the banks of the right superior temporal sulcus and progressive cortical thinning in the right medial orbitofrontal cortex in a synergistic way. MCI showed an interaction effect for both visual memory (Fig. 2; β for Aβ × Cortical thinning interaction = 3.18, t(50.8) = 2.79, *P* = 0.007, 95% CI based on bootstrap resampling = 0.975 ∼ 5.41), and verbal memory (β for Aβ × Cortical thinning interaction = 1.77, t(51.4) = 2.05, *P* = 0.045, 95% CI based on bootstrap resampling = 0.029 ∼ 3.47). That is, MCI patients with higher Aβ load in the right banks and faster cortical thinning in the right medial orbitofrontal cortex had faster visual and verbal memory decline. To serve as control analyses, we repeated the aforementioned analyses in the NCI group. NCI demonstrated longitudinal declines in visual memory (β for time = −0.069, t(10.8) = −3.47, P_FDR_ = 0.024, 95% CI based on bootstrap resampling = −0.108 ∼ −0.03) and verbal memory (β for time = −0.038, t(10.2) = −3.37, P_FDR_ = 0.024, 95% CI based on bootstrap resampling = −0.061 −0.016), similar to MCI. In contrast to MCI, there were no interaction effects on both memory measures between Aβ in the banks of the right superior temporal sulcus and progressive cortical thinning in the right medial orbitofrontal cortex (visual memory: *P* = 0.664, 95% CI based on bootstrap resampling = −4.72 ∼ 7.18; verbal memory: *P* = 0.165, 95% CI based on bootstrap resampling = −1.59 ∼ 8.37).

## Discussion

In this study, we retrospectively examined the global and regional relationships between cross-sectional amyloid deposition and longitudinal cortical thinning in individuals with clinically diagnosed MCI and NCI. There were no significant relationships between global Aβ burden and regional cortical thickness decline over time. However, elevated Aβ deposition in the right superior temporal banks was associated with a faster rate of cortical thinning in the right medial orbitofrontal cortex, which survived multiple testing correction. Notably, MCI patients with higher Aβ load in the right banks and faster cortical thinning in the right medial orbitofrontal cortex had greater visual memory decline longitudinally. Overall, these findings confer modest support to the notion of Aβ-related cortical thinning in non-local and select areas during the prodromal phase.

### Global Aβ burden is not associated with longitudinal regional cortical thinning

In our MCI cohort, we did not identify any strong relationships between cross-sectional global Aβ deposition and regional cortical thinning over time. The negative longitudinal findings corroborated and extended previous cross-sectional studies that similarly found null or weak effects of amyloid on regional brain atrophy.^31–35^ Moreover, tau may be the dominant driver or the mediator of some of the more subtle Aβ effects. Gordon and colleagues, for example, revealed that when both Aβ and tau PET values were combined in a model, increased tau uptake, but not Aβ, was associated with changes in cortical thickness.^31^ These findings corroborate the well-established spatiotemporal model of amyloid accumulation, in which Aβ levels are anticipated to have plateaued prior to symptom onset and macroscale neurodegeneration.^36^ Indeed, grey matter atrophy manifesting as cortical thinning is often considered a late event in the neurodegenerative cascade of AD. Cortical thinning is likely preceded by a multitude of cellular changes, such as neuroinflammation and tau, and so cannot be strongly explained by regional/local Aβ build-up. Furthermore, the significant time lag between the accrual of amyloid plaques and the onset of symptoms may have obscured any neurotoxic effects of amyloid. In contrast, the downstream hyperphosphorylation of neurofibrillary tau tangles and their spread from the medial temporal lobe to the neocortex is thought to be contingent on a crucial threshold of Aβ load and is frequently recognised to have stronger associations with clinical decline in AD.^37^ Our group and others have demonstrated strong links between regional tau deposition and cortical thinning in MCI and AD patients,^37–39^ supporting the idea that tau, not Aβ, is the primary driver of downstream neurodegenerative processes. Although our study primarily examined the associations between Aβ and subsequent cognitive decline and cortical atrophy, we acknowledge that tau pathology may play a significant role in mediating or influencing the effects of Aβ on these processes. Thus, it is essential to consider the potential interplay between Aβ and tau pathology in the context of our study results. Future research could address this issue by exploring the relationship between tau and cortical atrophy in a subgroup of individuals with mild cognitive impairment who test negative for amyloid. Such investigations will be crucial in elucidating the intricate mechanisms underlying AD pathophysiology and developing effective anti-AD interventions. We are currently collecting tau PET imaging, which may help us better understand potential interactions between Aβ and tau on regional cortical thickness (e.g. do people have tau-related cortical thinning only when they have a higher Aβ burden, and what are the corresponding spatial patterns? ).

### MCI with greater Aβ burden in the banks of the right superior temporal sulcus has greater cortical thinning in right medial orbitofrontal cortex over time

Using a ROI approach, we found that greater levels of Aβ deposition in the right superior temporal banks was associated with accelerated cortical thinning in the right medial orbitofrontal cortex in MCI. The involvement of temporal banks is particularly noteworthy. Recent research has shown that [^11^C]-PiB uptake in the banks of the superior temporal sulcus is the most Aβ-affected region in a cohort of cognitively normal adults, with greater sensitivity to identify early Aβ deposition than a composite measurement.^30^ While the lack of longitudinal [^11^C]-PiB data in our work precludes any analysis into how rates of Aβ accumulation may be related to trajectories of cortical atrophy, a prior study found that banks are among the areas with the greatest Aβ accumulation, as well as glucose hypometabolism and atrophy in autosomal dominant AD.^40^ Additionally, the superior temporal sulcus has shown early neuropathologic involvement (i.e. significant neuronal death)^41^ and might differentiate between people with moderate memory impairment who advance to AD and those who do not.^42^ Therefore, we hypothesise that the temporal banks are an early site of Aβ deposition, which might capture the early-stage effects of Aβ on cortical thinning prior to the onset of disease.

Notably, the right medial orbitofrontal cortex, which showed longitudinal cortical thinning in association with regional A deposition in the right banks, is also a site of early Aβ accumulation.^29^ This distant Aβ-atrophy association is consistent with a prior study, which revealed a cross-sectional correlation between frontal Aβ and temporal grey matter volume decline, however particular areas were not disclosed.^43^ To the best of our knowledge, the remote effects of Aβ on brain atrophy have not been studied extensively in preclinical or prodromal AD. While frontal cortical thinning is not a typical neurodegenerative hallmark of AD, previous research has highlighted its role in executive functioning.^44^

Previous research has demonstrated the distant effects of Aβ on neurodegeneration in AD.^45,46^ For instance, Aβ in frontal regions (as well as temporal and parietal regions) was related with extensive atrophy in previous studies using ^11^C-PiB, 18F-AV1451 and MRI data in healthy individuals.^47,48^ While the specific mechanisms behind the observed remote Aβ-atrophy relationship are still unclear, some of the associations may reflect distant effects of Aβ via prolonged deafferentation or increased tau aggregation in post-synaptic neurons connected by long-range white matter tracts. Within this framework, it is anticipated that amyloid plaque pathology would result in local synaptic deficits and, eventually, cell death in interconnected brain regions. In a sample of 61 MCI patients, the relationship of distant Aβ -cortical thinning was also reported in other DMN regions.^10^ Given the variability in clinical subtypes and disease progression, future research integrating Aβ PET, tau PET imaging, and diffusion weighted imaging (DWI) in larger samples of MCI patients will allow for additional fine-grained investigation into the pathological drivers underpinning brain atrophy during the prodromal phases.

### Interaction between Aβ and cortical thinning on visual memory decline in MCI

The clinical relevance of our findings could be evident through the significant interaction between banks Aβ and prefrontal cortical thinning on progressive visual memory decline in the MCI group. Specifically, MCI individuals with a combination of higher Aβ burden and faster cortical thinning showed greater visual memory decline. To the best of our knowledge, such interactions on visual memory decline has not been reported. However, a recent analysis of ADNI data has published Class II evidence implicating superior temporal banks Aβ burden in increased risk of cognitive decline amongst cognitively-normal elderly with nominal levels of Aβ.^30^ The mechanisms underpinning these interactions are still unclear, and larger cohorts are needed to facilitate the use of path analyses/structural equation modelling to disentangle the relative contributions of Aβ and cortical atrophy on visual memory decline. Longitudinal imaging and neuropsychology data with longer follow-ups of MCI to dementia conversion will be critical to further explore the clinical ramifications of our observed interaction. Should these findings be replicated in future studies, the combination of increased Aβ in the temporal banks and decreased prefrontal cortex thickness may constitute an emerging biomarker of incipient cognitive decline and be used as a marker to identify MCI persons at greater risks of a more aggressive disease course.

### Limitations and future directions

Our findings should be considered with several caveats. First, our sample size is relatively modest. It is possible that the clinical heterogeneity in MCI^49^ could have reduced our statistical power to detect other Aβ-associated cortical thinning. Future research with a larger sample size is required to corroborate our results, and to examine Aβ-atrophy associations along the disease spectrum and in MCI subgroups. The unavailability of tau-PET data for our subjects is another limitation since tau contributes to atrophy and may be partly dependent on supra-threshold levels of amyloid load.^50^ Furthermore, some of our participants may have concomitant pathologies, such as cerebrovascular disease. Future multimodal investigations are needed to investigate if and how different pathologies influence neurodegeneration in MCI. Finally, due to limited sample size of the NCI group (*n* = 12), our study was not statistically powered to draw conclusive results for the early preclinical stage. Further lifespan studies would be useful in determining longitudinal Aβ-atrophy trajectories from preclinical to prodromal and eventually the symptomatic phases. Finally, the absence of significant associations in our models may not necessarily indicate the absence of true effects. Future studies in larger cohorts with both tau and amyloid PET imaging, along with the use of structural equation modelling may be well-suited to elucidate these relationships further.

## Conclusions

Clarifying the relationships between Aβ accumulation and longitudinal cortical thinning at the early stages of AD has important implications for improving diagnostic and prognostic processes. In this longitudinal study, we found a non-local and statistically robust relationship between higher Aβ burden in the banks of the right superior temporal sulcus and greater longitudinal cortical thinning in the right medial orbitofrontal cortex in MCI exclusively, which was obscured when global Aβ was used. Additional multimodal investigations including information from other pathologies (e.g. tau and cerebrovascular disorders) are required to clarify the biological underpinnings and clinical implications of our findings.

## Supplementary Material

fcad192_Supplementary_Data

## Data Availability

Data used in this study are available upon reasonable request.
