# Supplementary material for: Longitudinal associations between β-amyloid and cortical thickness in mild cognitive impairment
Source: Brain Commun. 2023 Jul 4;5(4):fcad192. doi: 10.1093/braincomms/fcad192 (PMC10358322; doi:10.1093/braincomms/fcad192)
Supplement: fcad192_Supplementary_Data [file fcad192_supplementary_data.docx]

**SUPPLEMENTARY MATERIAL**


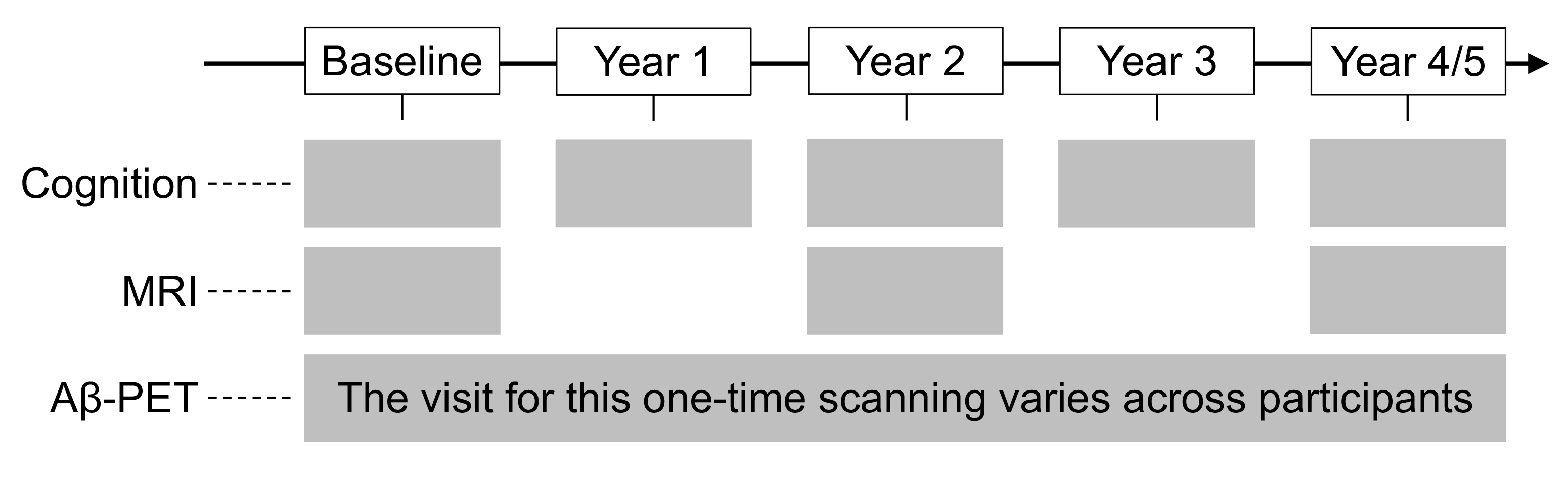


**Supplementary Figure 1. Schematic outline of the study design.** Recruitment is ongoing, which spans over 10 years. As such, not all participants have follow-up visits, or have the same number of follow-up visits. Cognition and MRI data are acquired between 2010 and 2020, and Aβ-PET scanning is conducted between 2016 and 2018.


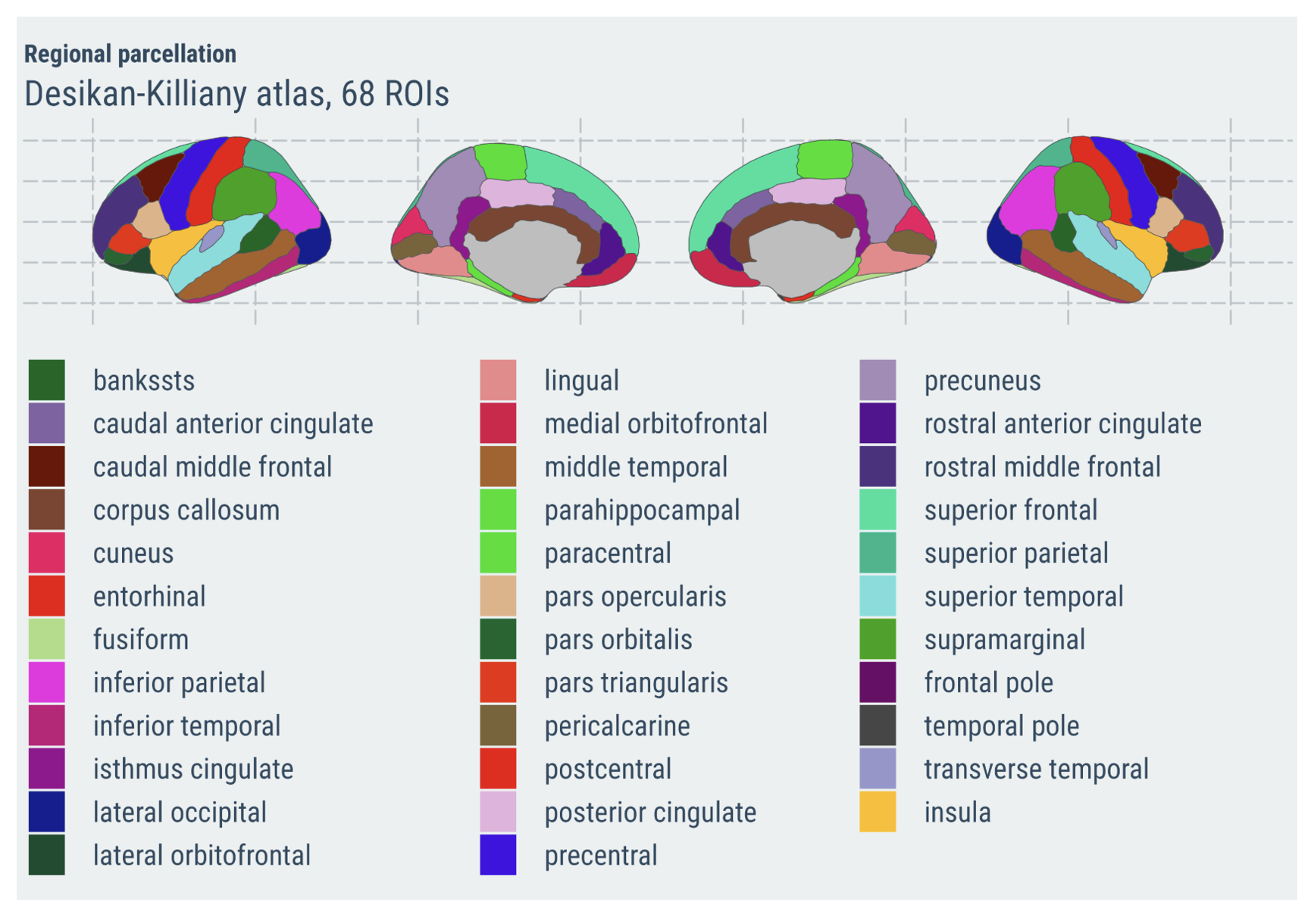


**Supplementary Figure 2. Illustration of regional parcellation using the Desikan Killany template.** Linear mixed models were built for MCI and NCI separately to evaluate the relationship between amyloid deposition and longitudinal cortical thinning across 68 ROIs, as defined by the Desikan Killiany atlas.
